# Difficulties with defining diagnostic accuracy study outcomes

**DOI:** 10.1186/1745-6215-16-S2-P49

**Published:** 2015-11-16

**Authors:** Katie Banister, Craig Ramsay, Jonathan Cook, Charles Boachie, Augusto Azuara-Blanco

**Affiliations:** 1 Health Services Research Unit, University of Aberdeen, Aberdeen, UK; 2 Nuffield Department of Orthopaedics, Rheumatology and Musculoskeletal Sciences, University of Oxford, Oxford, UK; 3 Robertson Centre for Biostatistics, University of Glasgow, Glasgow, UK; 4 Centre for Experimental Medicine, Queen's University Belfast, Belfast, UK

## Introduction

Evaluation of diagnostic tests raises unique methodological challenges. Outcomes include measures of test performance compared to a reference standard. When reporting diagnostic test accuracy, other factors to consider include the rate of indeterminate results and missing data [1]. However, there is little guidance on how this should be considered and represented within a diagnostic study.

## Methods

We conducted a paired study of the diagnostic accuracy of four imaging techniques for glaucoma. Participants were new referrals in UK secondary care. The reference standard was a clinical diagnosis of glaucoma by an experienced ophthalmologist.

Tests gave a glaucoma classification (outside normal limits, borderline, within normal limits) or were classed as indeterminate or missing. Analyses explored the causes of indeterminate results, alternative diagnostic scenarios including indeterminate results and alternative thresholds for the tests and reference standard.

## Results

943 participants were included in the analysis. Between 4 and 8% of imaging outputs were classed as indeterminate and this varied amongst imaging techniques. Indeterminate results were further classified into low quality result; no automated classification generated; imaging artefact; patient unable to undertake test.

## Conclusion

We used a generalisable systematic approach to considering categorisation and reporting of abnormal, indeterminate and missing test results. The handling of indeterminate results needs careful consideration during study conduct in order to inform decision making.

## Funding

NIHR HTA programme 09/22/111
